# The Transgenerational Transmission of Trauma: The Effects of Maternal PTSD in Mother-Infant Interactions

**DOI:** 10.3389/fpsyt.2020.480690

**Published:** 2020-11-30

**Authors:** Elisabetta Dozio, Marion Feldman, Cécile Bizouerne, Elise Drain, Mathilde Laroche Joubert, Malika Mansouri, Marie Rose Moro, Lisa Ouss

**Affiliations:** ^1^Action Against Hunger, Paris, France; ^2^Université Paris Nanterre, Nanterre, France; ^3^Hôpital Avicenne, Bobigny, France; ^4^Université Paris Descartes, Paris, France; ^5^Hôpital Cochin, Paris, France; ^6^Hôpital Necker-Enfants Malades, Assistance Publique Hopitaux De Paris, Paris, France

**Keywords:** trauma transmission, mother-infant relationships, cross modal interactions, PTSD (post-traumatic stress disorder), infants

## Abstract

The objective of the study was to examine the process of mother to infant trauma transmission among traumatized mothers in humanitarian contexts. We investigated the impact of mothers' post-traumatic stress disorder symptoms on the quality of the dyadic interaction by conducting a microanalysis of mother-infant interactions at specific moments when trauma was recalled, compared to more neutral moments. Twenty-four mother-infant dyadic interactions of traumatized mothers and children aged from 1.5 to 30 months Central Africa, Chad, and Cameroon were videotaped during three sequences: a neutral initial session (baseline) exploring mothers' representations of the infant and of their bonding; a second sequence, “the traumatic narration,” in which mothers were asked to talk about the difficult events they had experienced; and a third sequence focusing on a neutral subject. Three minutes of each sequence were coded through a specific grid for microanalysis [based on the scales developed at Bobigny Faculty of Medicine and the work of (1)], according to different communication modalities (touch, visual, and vocal), for both the mother and the child. Impact of traumatic event (IES-R), the level of depression and anxiety (HAD) were investigated in order to have a holistic understanding of the trauma transmission mechanism. The data analysis highlighted significant differences in mothers, children and their interaction during the “traumatic narration”: mothers touched and looked at the infant less, looked more absent and smiled less, and looked less at the interviewer; infants looked less at the interviewer, and sucked the breast more. The mother-child interaction “infant self-stimulation—mother looks absent” and “Infant sucks the breast—mother looks absent” occurred more often during the mothers' traumatic narrations. The “absence” of the mother during trauma recall seems to have repercussions on infants' behavior and interaction; infants show coping strategies that are discussed. We found no significant associations between interaction and infant gender and age, the severity of traumatic experience, mothers' depression and anxiety symptoms, and the country of residence. The results of the microanalysis of interaction can shed light on the fundamental role of intermodal exchanges between mother and infant in trauma transmission during mothers' trauma reactivation.

## Introduction

Trauma exposure is extremely common in countries affected by conflicts or natural disasters. In recent years, several studies have pointed to intergenerational trauma transmission (ITT) in countries affected by massive traumatic events (i.e., Burundi, Rwanda, Cambodia, Sierra Leone) and underlined the need to understand the mechanisms of transmission, in order to limit the potential negative impact on an entire community or even region, through generations (2–5).

Several studies in recent decades have tried to explain the transmission of parental trauma to the child. Many of these studies referred to collective historical trauma: the Holocaust, September 9/11, Vietnam Veterans, the Armenian genocide, etc., in which the impact of parents' post-traumatic stress disorder (PTSD) was measured in older children or in the second generation (6–10). Various other backgrounds have been explored in the process of ITT across generations, in different contexts of populations at risk for PTSD: low-income (11), abused mothers (12), adverse Childhood experiences (13) etc. Research has highlighted several possible models that may help explain the pathways of intergenerational trauma transmission.

Kellermann (14) proposed to distinguish the process of transmission, and what is transmitted. He described four models: psychodynamic, sociocultural, family system, and biological. Since this paper, two directions have been especially explored. Biological models have tried to understand the intergenerational transmission of stress (15) and the epigenetic mechanisms of stress and trauma transmission (16). Another set of studies concerns how the infants' sociocultural and family environment, the parents' representations, behavior and patterns of communication with their infant, can be considered as important factors involved in the process of ITT, a mechanism that appears to be a complex interaction between several co-processes [Perinatal Interactional Model of ITT, (17)]. Parental PTSD is characterized by physiological and emotional dysregulation, trauma related beliefs, and avoidance/withdrawal. This impacts fetal programming, and has repercussions on parent-child interaction through social learning, and maladaptive parenting. These phenomena decrease the child's regulatory capacity, increase child distress, and can lead to poorer child mental health, which in turn participates in a vicious circle.

The attachment theory (18) offers a theoretical framework to explain how caregiving behavior is impacted by parents' traumatic experience and can directly affect the child. Parents with unresolved trauma experience dissociation phenomena. They show frightened emotional expressions to the child who is unable to make the causal link between the terror of his/her parents and the trauma. The child responds with emotional and behavioral disorganization (19) and disorganized attachment behavior (20).

Maternal cognitions and representations also impact caregiving behavior, through reflective functioning. Schechter et al. (21) found that greater maternal PTSD severity was associated with unrealistic expectations of the child and distorted attributions of child intentions. These distorted maternal mental representations of the child (22, 23) will increase the frightening behaviors in response to the child, who consequently will react with disorganized behaviors. Child distress and reactions can reactivate traumatic memories in the mother, leading to a vicious circle. This can lead to specific offspring attachment orientations, in particular to higher attachment insecurities, either anxiety or avoidance, in association to self-amplifying cycle of PTSD and attachment insecurities of parents following trauma exposure (24).

Emotional availability (25) focuses more specifically on the emotional signaling between mother and child as a major mediator of trauma transmission. High scores indicate adequate maternal emotion regulation, and the open expression of negative emotions results in lower scoring. It has been proposed as a process involved in ITT, in spite of the fact that it was not found as a mediator between maternal trauma and infant psychosocial functioning in a sample of refugee mothers (26).

The PTSD mechanism was confirmed in a recent longitudinal study of ITT, which suggested a strong effect of maternal PTSD on the attachment relationship and consequently on child development; however, the need to identify the mechanisms by which maternal PTSD has an impact on the mother-infant relationship was underlined (27). The interpersonal neurobiological model of attachment and relational trauma developed by Schore (28, 29) can help to explain maternal PTSD transmission to the infant through a mechanism involving the rhythmic pattern behind mother-infant interactions (visual, tactile, vocal), which are negatively affected when mothers are traumatized. Mothers cannot modulate their stimulation and their response to their infant's needs, leading to overstimulation or on the contrary, to neglect of the child. Both behaviors can be traumatic for the infant who will have difficulty self-regulating his affective state to protect himself from becoming overwhelmed by hyperstimulation or the absence of stimulation from the mother. In a refugee sample, mothers' intrusion and avoidance explained individual differences in extremely insensitive parenting, which had direct negative effects on children's attachment organization (26).

In line with this mechanism, through the concept of “Affective Attunement,” Stern (30) proposed a pathway of transmission based on the sharing of emotional states between mother and child by means of inter-modal exchanges. D. Stern uses the term “intermodal” when in the dyadic exchanges an affect is expressed according to one modality (vocal, visual, bodily) then is “translated” and associated with another modality.

Affective attunement is a process centered on the mother's intermodal transformation of the baby's affective state. The mother tends to complement the baby's actions with gestures, gaze, and vocalizations, while the infant matches the mother's gesture behavior and perceives this as an affirmation of the continuity of his own affective state. The mother's transformation of the infant's affective state through behavior emphasizes the infant's recognition of his internal state. Traumatized mothers can transmit their negative affective states through their behavior, and these behaviors can modify the process of infant internalization of mothers' intentions.

More recently, a paradigm shift has been proposed for ITT in complex settings (war, migration). Intermediary dimensions have been introduced between parental PTSD and their effects on parent-child relationships. When parents were less able to draw on secure attachment representations, symptoms of PTSD increased the risk of insensitive parenting in a sample of Dutch asylum seekers and refugee parents (31). It is difficult to isolate the contribution of mothers' Post-Traumatic Stress Disorder (PTSD) in ITT, as in most cases, similarities with depressed or anxious mothers are frequent.

Studies of the direct mechanisms behind the transmission of parental traumatic experience to young children through caregiving have been undertaken recently, but are still rare (6, 20, 23, 26, 27, 32–35).

To investigate trauma transmission in a population at high risk of trauma exposure, we chose population from Central African Republic (CAR), which has been affected by collective violence since 2013, forcing the population to live in internal displaced camps or to migrate to refugee camps in border countries such as Chad or Cameroon. The current study addresses the issue of psychological trauma of the mother, which occurred in her life before the child was born. The objective was to identify the direct specific processes of mother-to-infant trauma transmission in traumatized mothers in humanitarian contexts, through the comparison of inter-modal mother-child interactions, occurring during different paradigmatic moments: moments of trauma recall (possibly leading to dissociation), compared to neutral moments, using a microanalysis method. We hypothesized that mothers affected by maternal post-traumatic stress symptoms would show lower levels of availability to respond to their infants' needs and show poor or inappropriate mother-infant interactions. When faced with the traumatic experiences or their recall (36), mothers may show specific behaviors (absent gaze, disconnection from reality, etc. as manifestations of dissociative states), which will result in a lack of attunement in interactions: non-synchrony (temporal coordination) or non-qualitative contingency (lack of inter-modality) between mother and infant behaviors. We hypothesized that during the narration of their traumatic experience in presence of the child, mothers may experience episodes of dissociation, that impact the infant's reactions and the interactions between mother and infant. Observing this specific moment in comparison with a neutral moment could highlight the specific mechanisms involved in trauma transmission.

## Methods

### Participants

Inclusion criteria: participants in the study were mother and child dyads in which the mothers had been exposed to traumatic events, according to criteria A of DSM V, in absence of the infant, or before the child's birth, including during pregnancy. Children were aged 1–36 months.

The recruitment took place in three African countries affected by the political crises in the Central African Republic (CAR), which started in 2013. In CAR, the sample comprised internally displaced mothers and their infants, whereas in Chad and Cameroon, the sample comprised refugees who had fled the ongoing violence in CAR.

The dyads were selected in accordance with the inclusion criteria by psychosocial workers working in the international NGO *Action Contre la Faim* (ACF) psychosocial support programs in the three countries. All the participants provided their written informed consent. Mothers were given the opportunity to ask questions to better understand the aims of the study before agreeing to take part and could refuse to continue the interview at any time.

The research protocol was approved by the institutional ethical review boards in CAR and in Cameroon, and by the “*Ministère de la Femme, de l'Action Sociale et de la Solidarité Nationale*” (Ministry of Women, Social Action and National Solidarity) in Chad.

### Procedure

The mothers were invited to participate in semi-structured interviews in the presence of their infant. The interview was videotaped to allow the microanalysis of mother and child behaviors and their interactions.

A clinical psychologist interviewed each mother with her infant in presence of the psychosocial worker who was in charge of the mother's psychological follow-up in the ACF program. This option was chosen in order to establish greater trust between the mother and the investigator and to guarantee a follow up in the case of increased distress in mothers and or infants during or after the research interview. The psychosocial worker was also the interpreter, as the investigator did not speak the local languages.

To investigate the core process of trauma transmission we developed a paradigm involving different settings, which explores the mother-infant interactions during three different moments: one moment of trauma recall, and two neutral moments, in order to compare within each dyad, each dyad being its own control, the changes in mother-child interaction.

- First sequence: a neutral initial session considered as the baseline to compare the other two sessions, “before the traumatic narration.” Some neutral questions explored mothers' representations of the infant and of their bonding,
- Second sequence: “the traumatic narration.” Mothers were asked to answer the question: “Could you tell me something about the difficult events you have experienced?” They were invited to narrate their traumatic events with the maximum of details (nature, temporality, numbers of events, etc.) and the effect on their present life (migration, change of situation, loss of family links, mourning, etc.). This was not the first time they narrated the events as they had already done so during the psychological follow up with the psychosocial worker.
- Third sequence: “after the traumatic narration,” began with the first mention by the mother of a neutral subject (i.e., the naming of the child, her projection into the future, cultural practices surrounding birth, etc.). The aim of these questions was to stabilize the mothers' emotional state.

### Measures

*Impact of traumatic events and Post-traumatic Stress Disorder (PTSD) symptoms* of mothers were screened using the Impact of Event Scale revised (IES-R) questionnaire (37). The scale consists of a list of 22 self-reported items assessing the perceived distress caused by traumatic events. Participants are asked to identify a specific stressful life event and then to say to what extent they had been affected during the past week on a 5-point Likert scale ranging from 0 “not at all” to 4 “extremely.” The IES-R total score ranges from 0 to 88 and subscale scores can also be calculated for the intrusion (8 items), avoidance (8 items), and hyperarousal (6 items) subscales. For each of these subscale scores, it is recommended to use means instead of raw sums. The maximum mean score on each of the three subscales is “4.” The IES-R is widely used for a preliminary diagnosis of PTSD (38). For the general IES-R score (calculated using summing), 33 and above represents the best cut-off for a probable diagnosis of PTSD (39). The literature shows that even though not formally validated in all the different contexts, the IES-R scale is used to measure PTSD symptoms in many cultures throughout the world (40). For this study, the French version of the IES-R was used, which has satisfactory internal validity and test-retest reliability (41).

*Symptoms of depression and anxiety* were measured using the Hospital Anxiety and Depression scale (HAD) (42). The HAD is an instrument for symptoms related to anxiety (HADS-A) and depression (HADS-D) composed of 14 items. Each question asks about the frequency of specific symptoms in the past week using a 4-point scale ranging from 0 (*not at all*) to 3 (*very often*). For each subscale, the cut-off identified is a point of 8/21. This instrument has been validated in both medical and general populations, and the results of a recent systematic review on screening tools for common mental disorders in low and middle income countries (43) broadly recommend using the HAD scale for depressive and anxiety disorders, as it been validated in multiple settings.

#### Microanalysis of Mother and Child Behaviors

We chose to conduct a microanalysis of the mother-child interaction in order to identify instant-by-instant events and the intermodality of interaction. Videotaped mother and child interactions during the interview were analyzed with an observation grid based on the Action Research Training (RAF, *Recherche Action Formation*) scales developed at the Bobigny Faculty of Medicine (44) and on the microanalysis conducted by Beebe et al. (45) on mother-infant interactions (Table 1). These previous studies analyzed mother/infant or mother/toddler behaviors according to the different types of communication: tactile, vocal and visual.

**Table 1:** Observation grid for mother and infant communication modalities.

**Modality of communication**	**Behavior observed**	
	**Mother**	**Infant**
Bodily	Touches the infant Touches an object	Touches the mother Touches an object Self-stimulation/ self-regulation Sucks the breast Sleeps
Visual	Looks at the infant Look at what is doing the infant Looks at the interviewer Look at the environment Looks absent	Looks at the mother Looks at the interviewer Look at the environment Looks at an object Looks at his body Looks absent
Vocal	Speaks to the infant Vocalizes to the infant Speaks to the interviewer	Vocalizes Vocalizes to the mother Vocalizes to the interviewer
Affects	Smiles Smiles at the infant Cries	Smiles Smiles at the mother Cries

In addition to the distinct behavior and communication actions of mother and child, a list of possible communication overlaps, which could suggest a possible interaction, was defined. Based on the most frequently observed interactions, 28 mother-child communication modality pairs were retained for the analysis (Table 2).

**Table 2:** Grid for microanalysis of mother-infant interactions.

**Mother–infant interactions**
Infant touches the mother - Mother touches the infant
Infant touches the mother - Mother looks at the infant
Infant self-stimulation - Mother looks absent
Infant sucks the breast - Mother touches the infant
Infant sucks the breast - Mother looks at the infant
Infant sucks the breast - Mother looks absent
Infant looks at the mother - Mother touches the infant
Infant looks at the mother - Mother looks at the infant
Infant looks at the mother - Mother looks absent
Infant look at an object - Mother looks at what is doing the infant
Infant looks absent - Mother touches the infant
Infant looks absent - Mother looks at the infant
Infant looks absent - Mother looks absent
Infant vocalizes - Mother vocalizes to the infant
Infant vocalizes - Mother speaks to the infant
Infant vocalizes - Mother touches the infant
Infant vocalizes - Mother looks at the infant
Infant vocalizes - Mother looks absent
Infant smiles - Mother touches the infant
Infant smiles - Mother looks at the infant
Infant smiles - Mother looks absent
Infant smiles at the mother - Mother miles at the infant
Infant cries - Mother touches the infant
Infant cries - Mother looks at the infant
Infant cries - Mother looks absent
Infant cries - Mother smiles

We selected the first 3 min of each of the three moments of videotaped interview (before, during and after the “traumatic narration”) for analysis.

In each sequence of 3 min, any change in behavior, among those listed in the grid shown in Table 1, was noted and measured in terms of duration. Interrupts of the same action lasting <0.50 s were not taken into account. The action was then coded as continuous. “Touch” was coded as such only if the gesture was intentional. Contacts between mother and baby caused by unintentional gestures were not considered. The fact that the child was sitting on the mother's lap was not taken into account in intentional tactile interactions.

The frequency and the duration of the mother-infant inter-modal interactions were coded using the open source multimedia annotator Elan, version 4.9.3 (46). The software was used to code the length of intermodal distinct behavior and interactions. Communication overlaps (see Table 2) were generated automatically by the Elan software. Inter-rater reliability (Cohen's Kappa) of the coding of mother and infant behaviors was calculated on a randomly selected sample of 25% of the dyads interviewed and was satisfactory (*k* = 0.686).

### Statistical Analysis

Friedman's test was used for the analysis of difference in behaviors and interactions before, during, and after the traumatic narration by the mother.

The choice of a non-parametric test was made because of the small sample size. Among the non-parametric tests, Friedman's test is an ideal statistic to use for a repeated measures type of experiment to determine if a particular factor has an effect (47). The Friedman test is a non-parametric alternative to the repeated measures ANOVA when the assumption of normality is not acceptable.

In addition, to examine where the differences actually occurred, we ran separate Wilcoxon signed-rank tests on the different combinations of related groups. Spearman's correlation was used to analyse correlations between scale scores and demographic variables. All statistical analyses were performed using SPSS version 21.

## Results

### Descriptive Results

Twenty-four mother and infant dyads met the inclusion criteria of this study. Sixteen were recruited in CAR, three in Chad and five in Cameroon. The demographic data for the 24 dyads are presented in Table 3.

**Table 3:** Demographic data of participants.

**Dyad**	**Country**	**Age of the mother (years)**	**Age of the child (months)**	**Sex of the child**	**Number of children in the family**	**Type of traumatic event**	**Mother : pregnant during the traumatic event**	**Presence of the father**	**Raison of the absence of father**
1	Cameroon	35	11	F	7	1;2;4;5	Yes	Yes	–
2	Cameroon	30	7	F	3	1;2;3;4;8	Yes	Yes	–
3	Cameroon	25	1,5	F	6	1;2;3;4	No	Yes	–
4	Cameroon	26	12	F	MD^*^	1;2;3;5	Yes	Yes	–
5	Cameroon	MD	6	F	MD	1;2;3;6;7	Yes	No	Disappeared
6	CAR	18	9	M	1	1;3;5;7	Yes	No	Disappeared
7	CAR	28	30	M	5	1;3;4	Yes	Yes	–
8	CAR	37	7	F	4	1;3;4;8	Yes	Yes	–
9	CAR	MD	17	M	1	1;3;4;6	No	Yes	–
10	CAR	MD	17	M	?	1;3;4	Yes	Yes	–
11	CAR	19	8	F	1	1;3;4;7	Yes	No	Disappeared
12	CAR	MD	12	F	MD	1;3;4;6	Yes	No	Dead
13	CAR	16	12	M	1	1;3;4	Yes	Yes	–
14	CAR	26	11	M	2	1;3;6	Yes	Yes	–
15	CAR	31	24	M	3	1;3;4	Yes	Yes	–
16	CAR	28	14	F	3	1;3;4;5;6	Yes	Yes	–
17	CAR	16	7	F	1	1;3	Yes	Yes	–
18	CAR	20	8	F	2	1;3;4;5;6;7	No	No	Disappeared
19	CAR	28	7	M	2	1;3;4	Yes	Yes	–
20	CAR	29	28	M	9	1;3;4	Yes	Yes	–
21	CAR	32	10	M	6	1;3;4;5	Yes	No	Dead
22	Chad	MD	12	F	3	1;3;4	No	No	He stayed in CAR
23	Chad	MD	8	F	MD	1;3;4	Yes	No	He Fled
24	Chad	25	8	M	1	1;3;4;5	Yes	No	Dead

* *MD, Missing Data*.

*1 Escape from gunfire, detonations, violence*.

*2 Migration on foot in extremely difficult conditions*.

*3 Exposure to combat and violence*.

*4 Witness to the murders*.

*5 Witness to the death of a family member*.

*6 Death of loved one due to the conflict*.

*7 Husband's disappearance*.

*8 Death of his own child(ren)*.

The children's age ranged from 1.2 to 30 months [*M* = 11.94, *SD* = 6.92; Median = 10.55, Interquartile Range (IRQ) = 6.3] and consisted of 11 boys and 13 girls.

The mothers were aged between 16 and 37 [*M* = 26, *SD* = 16.18; Median = 27, Interquartile Range (IRQ) = 11]. Some mothers were unable to indicate their own age, as they did not know it. This is quite frequent in the rural contexts of countries in the field of this study. Eighty-three percent of the mothers were pregnant during the traumatic event; four children were born during traumatic situations. Only one mother was pregnant during the interview. At the time of the interview, all of the children were the youngest of the siblings. The father was present in 62.5% of the cases. His absence was due either to his death or to his disappearance, without having given any news.

Mothers had experienced multiple traumatic events. The detail for each mother is shown in Table 3: among the traumatic events they had witnessed the murder of their children, or husband, relatives or friends, some of them witnessed massive violence in the community and/or had been forced to leave their homes, lost all their possessions, and feared for themselves and their loved ones, etc. The time between the traumatic event and the interview was on average 16 months, with a minimum of 7 and a maximum of 31 months (Median = 13, IRQ = 11).

As shown in Table 4, the impact of the traumatic event was very high among mothers. In addition, they presented high rates of depression and anxiety.

**Table 4:** Clinical status.

**Measure**	**Outcome**	**Min**	**Max**	***M***	***SD***	**Median**	**IRQ**	**Range**
HAD	Anxiety	5	18	13.08	3.425	14	4.5	Clinical
	Depression	8	17	12.33	2.180	12	3	Clinical
IES-R	Avoidance	1	3	1.96	0.374	1.85	0.7	High
	Intrusion	1	3	2.36	0.687	2.55	2.6	High
	Hyperarousal	0	3	2.22	0.817	2.25	1.2	High
	Total PTSD	20	64	47.08	10.685	49.5	12.8	Clinical

### Correlations Between Clinical Status and Demographic Variables

Correlations were run to assess the relationships between the clinical status (scores of HAD and IES-R scales in Table 4) and the main demographic variables reported in Table 3. Spearman's correlation coefficient was used rather than the Pearson correlation coefficient because of the small sample size (*n* = 24).

No correlation was found between scores at HAD or IES-R and the age of the mother, the gender and age of the child, the presence of the father, the fact of being primiparous and the number of children.

Results of the Spearman correlation indicated that there was a significant positive association between the severity of the impact of the traumatic event (IES-R) and the time that had elapsed since the traumatic events (*r* = 0.54, *p* = 0.01), meaning that the more time had passed since exposure to the traumatic event, the more the participants showed traumatic symptoms, in particular for intrusion (*r* = 0.45, *p* = 0.05) and hyper arousal (*r* = 0.468, *p* < 0.001), both measured through IES-R subscales.

The Wilcoxon-Mann-Whitney test was used to cross the qualitative variables with two classes such as the gender of the child, with the quantitative variable of the clinical scales. No significant differences were found between the gender of the child and the clinical profile of the mother.

A Kruskal-Wallis test was conducted to examine the differences in mothers' clinical symptoms according to the country of residence. Results indicated that the highest level of PTSD symptoms was associated with populations still living in CAR, H (2) = 8.5, *p* = 0.014.

No other clinical symptoms (Anxiety and Depression) were linked to the Country of residence.

### Results of Microanalysis

Results refer to separate actions by the mother and by the infant, and to their interactions.

#### Differences in the Mothers' Behaviors Between the Three Sessions

Analysis revealed the following statistically significant differences (Table 5): the mother spoke to the interviewer (*p* = 0.030) more during the traumatic narration than in the session after (*Z* = −2.171, *p* = 0.030); the mother touched the infant (*p* = 0.001) less during the traumatic narration than in the session before (*Z* = −3.589, *p* = 0.000) and more than in the session after (*Z* = −2.886, *p* = 0.004); the mother looked at the infant (*p* = 0.018) less during the traumatic narration than after (*Z* = −3.494, *p* = 0.000); the mother looked at the interviewer (*p* = 0.006) less during the traumatic narration than before (*Z* = −2.886, *p* = 0.004) and less than in the session after (*Z* = −2.800, *p* = 0.005); the mother looked absent (*p* = 0.004) more during the traumatic narration than before (*Z* = −2.950, *p* = 0.003) and more than after (*Z* = −3.346, *p* = 0.001); the mother smiled (*p* = 0.004) less during the traumatic narration than before (*Z* = −2.675, *p* = 0.007) and after (*Z* = −2.045, *p* = 0.041). No other significant differences were found.

**Table 5:** Differencesin the duration of the mothers' behaviors in the three sessions.

**3 min session**					**3 min session**				**3 min session**				**Asymp**	**Pairwise comparison**
**before (a)**					**during (b)**				**after (c)**				**Sig.**	**(Wilcoxon sing-ranked test)**
	**Min**	**Max**	**Mean**	**MD**	**Min**	**Max**	**Mean**	**MD**	**Min**	**Max**	**Mean**	**MD**		
			**(SD)**	**(interquartile range IQR)**			**(SD)**	**(IQR)**			**(SD)**	**(IQR)**		
**Vocal interactions**														
Mother speaks to the infant	0	1.1	0.08 (0.28)	0 (0)	0.8	2.3	0.18 (0.55)	0 (0)	0.3	16.6	1.37 (3.46)	0 (1.9)	*0.139*	
Mother vocalizes to the infant	0	6.3	2.61 (1.27)	0 (0)	0	0	0 (0)	0 (0)	0	0	0 (0)	0 (0)	*0.368*	
Mother speaks to the interviewer	26.4	136.7	65.23 (28.43)	58.2 (35.7)	30.03	180.7	75.02 (35.25)	63.8 (22.9)	0	104.6	55.85 (25.79)	53.1 (36.6)	0.030^*^	***b* > *a*;** *b* > c^*^
**Touch interactions**														
Mother touches the infant	0	152.7	58.34 (47.07)	46.1 (79.2)	0.1	118.3	23.35 (28.71)	14.7 (-23)	0.2	164.2	52.13 (40.46)	47.2 (67.6)	0.001^*^	*b* < a^*^; *b* < c^*^
Mother looks at the infant	0	20.03	6.57 (6.83)	3.9 (13.4)	0	14.7	3.35 (4.45)	1.6 (5.7)	0	45.1	10.68 (11.79)	7.1 (9.5)	0.018^*^	*b* < *a*; *b* < c^*^
Mother looks at what the infant is doing	0	46.9	8.69 (10.93)	4.9 (13.2)	0	20.6	3.66 (5.31)	1.7 (5.9)	0	39.8	6.75 (10.26)	2.2 (9)	*0.096*	
Mother looks at the interviewer	23.8	178.5	114.95 (28.12)	11.5 (48.3)	19.7	169.6	90.69 (39.53)	91 (57.1)	22.9	156.9	106.82 (37.43)	115.6 (55.6)	0.006^*^	*b* < a^*^; *b* < c^*^
Mother looks at the environment	0	73.1	14.06 (15.55)	9.9 (13.3)	0	98.3	18.37 (26.4)	8.2 (19.5)	0	107.9	18.07 (23.61)	9.8 (16)	*0.153*	
Mother looks absent	0	145	34.78 (37.43)	22.3 (34.1)	0	146	59.17 (44.1)	50.9 (74.6)	0	119	36.8 (32.12)	25.6 (35.3)	0.004^*^	*b* > a^*^; *b* > c^*^
**Affects**														
Mother smiles	0	57.9	13.0 (18.26)	4.04 (17.4)	0	47.6	3.59 (10.63)	0 (0.5)	0	77	12.15 (21.13)	3.25 (16.6)	0.004^*^	*b* < a^*^; *b* < c^*^
Mother cries	0	0	0 (0)	0 (0)	0	88.2	3.67 (18)	0 (0)	0	0	0 (0)	0 (0)	*0.368*	

* *p < 0.05*.

#### Qualitative Analysis of Mothers' Behavior

The mothers' vocal interactions with the infant were very limited; mothers were more involved in speaking with the interviewer, especially when they were recounting the traumatic event. The mothers' visual interactions with the infant were poor and more focused on the interviewer.

All the mothers touched the infant at least once during one of the three sessions analyzed. Most of the time during the interview the mother showed a neutral facial affect except for a visible change of gaze when narrating the traumatic event. Only one mother cried and 17 mothers smiled at least once, but they never smiled at the infant.

#### Differences in the Infants' Behaviors Between the Three Sessions

The repeated measures compared using Friedman's test revealed a statistically significant difference in (Table 6): “Infant looks at the interviewer” (*p* = 0.001) and “Infant sucks the breast” (*p* = 0.045). *Post-hoc* analysis with Wilcoxon signed-rank tests was conducted and showed that the infant looked at the interviewer less during the mother's traumatic narration than in the session before (*Z* = −3.360, *p* = 0.001) and they sucked the breast more during the mother's traumatic narration than before (*Z* = −2.395, *p* = 0.017). No other changes in infant behavior were significant in the three sessions.

**Table 6:** Differences in the duration of infants' behaviors in the three sessions.

**3 min session**					**3 min session**				**3 min session**				**Asymp**	**Pairwise comparison**
**before (a)**					**during (b)**				**after (c)**				**Sig.**	**(Wilcoxon sing-ranked test)**
	**Min**	**Max**	**Mean**	**MD**	**Min**	**Max**	**Mean**	**MD**	**Min**	**Max**	**Mean**	**MD**		
			**(SD)**	**(interquartile range IQR)**			**(SD)**	**(IQR)**			**(SD)**	**(IQR)**		
**Vocal interaction**														
Infant vocalizes	0	69.2	6.24 (14.94)	1.51 (5.1)	0	70.3	10.85 (18.35)	0.85 (11.4)	0	35.1	7.29 (10.09)	1.25 (13.3)	0.532	
**Touch interactions**														
Infant touches the mother	0	98.7	27 (30.26)	11.51 (45.2)	0	174.3	44.21 (43.87)	31.15 (55.1)	0	167.4	46.2 (49.03)	28.8 (49.3)	0.158	
Infant touches an object	0	180.4	95.73 (68.88)	88.49 (145.5)	0	180.7	83.61 (71.66)	82.5 (152.6)	0	180.8	58.21 (66.89)	31.5 (123.4)	0.071	
Infant self-stimulation	0	179.7	21.18 (40.29)	4.03 (28.8)	0	151.2	29.45 (41.31)	9 (38.9)	0	173.1	25.21 (498)	2.65 (27.5)	0.542	
Infant sucks the breast	0	180.0	16.45 (42.76)	0 (0)	0	180.8	50.72 (71.58)	0 (100.3)	0	152.8	27.81 (51.53)	0 (42)	0.045^*^	*b* > a^*^; *b* > *c*
**Visual interactions**														
Infant looks at the mother	0	29.1	2.47 (6.12)	0.2 (1.7)	0	10.2	1.28 (2.5)	0 (1.6)	0	15.2	3.7 5 (4.71)	1.65 (5.7)	0.288	
Infant looks at the environment	0	131	39.15 (39.37)	31.8 (56)	0	122.2	43.42 (34.39)	42.85 (44.9)	0	104.2	36.32 (30.72)	27.9 (62.8)	0.722	
Infant looks at the interviewer	0	103.7	37.34 (27.94)	30.29 (31.2)	0	79.2	16.45 (20.56)	7.3 (24.3)	0	71.9	16.23 (20.1)	10.2 (26.5)	0.001^*^	*b* < a^*^; *b* > *c*
Infant looks at his own body	0	28.5	3.25 (6.93)	0 (3.3)	0	20	1.76 (4.39)	0 (1.9)	0	41.8	2.5 (8.7)	0 (0)	0.195	
Infant looks absent	0	64.4	24.78 (21.04)	20.4 (38.1)	0	86.9	24.78 (30.16)	10.25 (43)	0	78.9	18.59 (26.46)	3.15 (30.8)	0.364	
**Affects**														
Infant smiles	0	7.5	1.28 (2.25)	0 (3.2)	0	3.5	0.73 (1.17)	0 (1.4)	0	19.9	1.85 (20.06)	0 (2)	0.980	
Infant cries	0	18.1	1.8 (4.97)	0 (0)	0	57.3	4.99 (12.92)	0 (1.2)	0	86.5	8.4 (20.5)	0 (1)	0.412	

* *p < 0.05*.

#### Qualitative Analysis of Infants' Behavior

As with the mothers, very few *vocal* infant interactions were observed during the sessions. Vocalizations were generally neutral or positive. To determine the direction of the infant's vocalization, we observed the direction of gaze and / or the spatial position of the infant's body, to understand if the vocal interaction was toward the mother and/or the interviewer. Infants produced no vocalizations toward the mother or the interviewer.

The infants' *visual* interactions tended to be directed toward an object: a toy, the chair where they were seated with their mother, objects on the ground, etc. When the infant changed the direction of his gaze, it was toward the interviewer or the environment, rather than toward the mother. Touch was the most frequent communicative means used by infants. All the infants touched their mother at least once during the three sessions. In particular, the tactile interaction was more frequent during the traumatic narrative. The majority of children (21 of 24) showed self-stimulation behaviors. We included in self-stimulation broader behaviors represented by the repetition of physical movements (e.g., repetitive swaying, movement of the fingers, hands, etc.), which can be self-regulation behaviors.

Sucking the breast was included among the physical interactions and 11 infants suckled at least once during the three sessions. These infants were aged from 1.5 to 17 months (*M* = 11.5, *SD* = 4.47; Median = 12, IRQ = 4). In general, infants showed a neutral expression during the sessions. One third expressed affect by smiling or crying.

#### Differences in the Mother-Infant Interactions Between the Three Sessions

The repeated measures of all the 28 interactions between mother and child revealed that only two variables were statistically significant: “Infant self-stimulation—Mother looks absent” (*p* = 0.045) that occurred more during the mother's traumatic narration than in the session before (*Z* = −2.999, *p* = 0.003) and more than in the session after (*Z* = −2.731, *p* = 0.006). “Infant sucks the breast—Mother looks absent” (*p* = 0.003) occurred more during the traumatic narration than before (*Z* = −2.090, *p* = 0.037) and more than after (*Z* = −2.118, *p* = 0.034).

Correlations were run to assess the associations between the mother-infant interactions and demographic variables such as age and gender of infant, age of the mother, presence of the father, etc., and clinical status of the mothers (HAD and IES-R scores). No significant associations were found.

#### Qualitative Analysis of Mother-Infant Interactions

The analysis of mother and infant interactions showed very little reciprocity and contingency of interactions. Interaction in the same modality: “infant touch—mother touch,” “infant vocalization—mother vocalization or speaking,” “infant looks at the mother—mother looks at the infant,” were rare.

## Discussion

The purpose of this study was to shed light on the process of mother to child trauma transmission in mothers who had experienced severe traumas in humanitarian contexts. As far as we know, this is the only study to investigate the quality of the dyadic interaction, by observing and conducting a microanalysis of the mother-infant interactions in a trauma reactivation situation. The mother-child interaction during a trauma recall situation was compared with mother-child interaction during a more neutral interview.

We found three main results, confirming our hypothesis.

The first result was that the mothers' behavior toward the child was specifically affected during trauma recall narration compared to the two more neutral moments of interaction. During the “traumatic narration,” mothers appeared to be emotionally affected by memories: they touched the infant less than in the session before, they looked at the infant less than in the session after, they looked more absent and smiled less than “before” and “after” moments. Moreover, they looked at the interviewer less during the traumatic narration than before and after. This is in favor of the relevance of our setting, which tried to catch these dissociative moments. When they were faced with the trauma recall, the mothers' absent look suggested that they were re-experiencing the event and were unable to focus on the present situation and on the infant's requests. They showed a behavioral pattern linked to the traumatic experience: they withdrew from interaction, smiled, and looked less at others, and seemed to be absent. These signs may reflect the maternal traumatic dissociation the infants face when their mother is lost in the traumatic experience. The mothers seem cut off from the present experience without being able to perceive external reality and the needs expressed by their child (48). This suggests that the mothers had difficulty in properly assessing the verbal and non-verbal expressions of infant arousal because they were overwhelmed by their own emotional state. This “absence of an appropriate response” emphasizes the transmission of the mothers' negative emotional state to the infants, who can potentially internalize this affective state as their own (30).

The majority of children in this study had no access to the direct meaning of their mother's words as they still were in a pre-verbal developmental stage, and even for those aged between 12 and 30 months, their mothers' words could not attack their symbolic world since they were not yet able to organize Internal Working Models, due to their developmental stage (49). Consequently, what is traumatic for them is more related to the absence, the lack of response to their requests and queries, which can potentially create external and internal sensory chaos (50).

The second finding is that the infants' behavior was impacted by their mothers' traumatic experience. During the traumatic narration, infants looked at the interviewer less than in the session before, and they sucked the breast more than before. Moreover, the mother-child interaction was also affected: “Infant self-stimulation—Mother looks absent” and “Infant sucks the breast—Mother looks absent” occurred more during the traumatic narration than in the other two sessions. These interactive behaviors reflect the children's coping strategies of self-regulation: they perceive these moments, and try to compensate with self-stimulation or by clinging to the satisfaction of primary needs. To suck the breast is a behavior which maintains close contact with the mother, and offers a primary satisfaction that the child does not find in the interaction. The lack of correlation between the age of the child and suckling the breast suggests that in the case of older infants (more than 12 months), this request was not simply linked to the need to be fed, but to a possible coping strategy for self-regulation in a stressful situation. These two coping strategies are efficient in providing self-comfort and limiting the negative effect of distress (51). This is in line with studies by Tronick (52), who stated that infant self-stimulation is a coping behavior that reduces stressful situations and offers self-calming, in particular when mothers are “still faced” (53). The mothers' “absent look” is a kind of ecological “still face” situation. These interactive behaviors reflect the children's coping strategies of self-regulation: they perceive these moments, and try to compensate with self-touch or by clinging to the satisfaction of primary needs. A previous research has shown that during a still-face procedure, when mothers were unavailable, infants spent more time touching themselves, supporting the regulatory and exploratory roles of infant touch, especially during periods of maternal unavailability (54).

It should be noted that, according to Main and Solomon "Indices of Disorganization and Disorientation (Main and Solomon, unpublished manuscript), the children did not show disorganized behavior during their mothers' “absence,” but rather showed adaptive behaviors such as self-stimulation. Several explanations can be put forward for this. First, mothers had received psychological care from NGO psychosocial workers, which had possibly helped them to reduce their traumatic experience; our paradigm of trauma recall may perhaps not have reflected the fact that their trauma was reduced. Second, infant self-stimulation may be the first stage of coping; if the mother's “absence” is repeated, their adaptive strategies may prove insufficient to handle the mother's negative experience and may lead to internalized working models of disorganization. Third, the fact that the mothers talked very little to their child could limit the trauma transmission. Last, cultural differences might explain the way they cope with traumatic experiences. In traditional societies, children are not only raised by their mothers (55). They grow up surrounded by several caregivers, who complete the construction of the world and the child's identity. This aspect of shared mothering could be a protective factor in the transmission of trauma from mother to child. The presence of a co-mothering system could mitigate or compensate for the effects of the mother's lack of response to the child's requests when she is absorbed by traumatic intrusions.

The third finding was that there were no significant associations between interaction and infant gender and age, the severity of traumatic experience assessed by IES, and mothers' depression and anxiety symptoms assessed by HAD. This is maybe due to the small sample. Concerning age, it has however been shown (34) that the period when child locomotion develops might represent a critical time window for mother-child interaction in dyads with mothers having a history of abuse.

Not even the country of residence had an influence on the interactions between the mother and infant. The only difference was linked to the impact of the traumatic event and the dyads' country of residence. The highest level of PTSD symptoms was associated with those living in the Central African Republic (CAR). This can be explained by the fact that people living in CAR are in an ongoing extremely traumatogenic context, whereas people who migrated and are living in refugee camps are more protected against new traumatizing situations.

Despite the fact that we found a significant positive association between the severity of the impact of the traumatic event (IES-R) and the time that had elapsed since the traumatic events, neither the severity of the traumatic experience nor the severity of anxiety or depression were associated with interactive behaviors. This may indicate that among the war traumatized and refugees, the theoretical frame to understand ITT has to be enlarged. Mothers' general psychological distress, but not PTSD, was directly associated with negative parenting and child psychosocial difficulties in a sample of 291 Syrian refugee mothers in Lebanon who had been exposed to war trauma in the past (56). These results argue in favor of taking into account a broader framework for complex trauma in conflict and post-conflict areas, including the psychosocial framework, with the trauma focused approach. We did not evaluate the impact of other psychosocial conditions.

## Limitations of the Study

The main limitation of the study is the size of the sample. The recruitment in humanitarian contexts involves several challenges linked to security constraints and difficulties of access to subjects. A larger sample would be more representative of the observed mechanisms of transmission and make our results more significant. However, even with the small sample, our results point to particular lines of enquiry to pursue in future studies. The cultural particularities of mother and child relationships require investigation to design an appropriate framework for analysis as a key to understanding the affection linked to behaviors and interactions. The second main limitation is the absence of a control group. But our aim was to explore how trauma recall can affect mothers' presence to the child and the way they interact with the child, and not to globally qualify the interactive mother-child process. That is why we compared each dyad in three different settings, each dyad being its own control. A control group could have allowed us to compare mother-child interaction in play sessions. But by definition, it is not relevant to explore trauma recall with mothers who have not experienced traumatization. Another limitation is the context in which interaction took place, which was set up to catch the direct effects of trauma reactivation. The mothers were probably more involved in the interview than in playing with the child; however, this setting was close to an ecologic situation of daily life. Future studies should include longitudinal observation of mother and child dyads, with control groups, to identify the long-term impact of mothers' inappropriate response impacted by dissociation, when trauma is recalled, to their infants' needs and child development.

## Conclusion

This research is unique in its approach to mother-infant interactions: in the field explored (ITT in humanitarian contexts), by the setting (trauma recall), and by the microanalysis of interaction. Our results shed light on the process of trauma transmission, focusing on the caregiver reactions during trauma recall, and suggest that trauma affects a mother's availability to interact with her infant and to regulate his/her state of emotional arousal. The infant experiences this sudden lack of proper responses from the mother when she faces events or thoughts that trigger trauma memories, which can have an impact on the infant's perception of his/her own emotional status. A new and surprising finding was that children did not react by disorganization as has been previously described, but with self-stimulation, representing possible coping strategies in reaction to the mother's enigmatic reaction. This adaptive behavior may compensate for the mothers' psychic absence and for the lack of “attunement” between mother and child.

Our results strongly reflect the increasing need to understand trauma transmission mechanisms in highrisk populations, particularly when they live in unremitting traumatogenic conditions.

In particular, the question about self-stimulation during mother's “absence” should be considered in further longitudinal studies as this initial adaptive mechanism could lead to internalized working model of disorganization, if repeated.

To prevent or to limit the impact of maternal trauma transmission, we recommend psychological interventions with mothers, starting from their pregnancy, and continuing after childbirth during the important period for the development of the child, until 2 years. Psychological support for at-risk mothers should aim to allow them to resolve the effects of the trauma in order to limit the transmission of trauma in interactions with the child. Interventions focusing on parental skills, their sensitivity, their ability to decipher, and respond to the specific needs of their child like video feed back interventions (57) are also recommended. But psychological support, which has been proposed for mothers with children born of sexual violence (58), has to go beyond trauma focused interventions, and provide a holistic and community embedded approach that can be applied across settings.

## Data Availability Statement

The datasets generated for this study are available on request to the corresponding author.

## Ethics Statement

The studies involving human participants were reviewed and approved by the institutional ethical review boards in CAR and in Cameroon, and by the Ministère de la Femme, de l'Action Sociale et de la Solidarité Nationale in Chad. Written informed consent to participate in this study was provided by the participants' legal guardian/next of kin.

## Author Contributions

EDo was the principal investigator of this research and she was supervised by MF, MMo, CB, and LO during data collection and analysis. EDo, MMa, MMo, EDr, ML, and LO all contributed to the definition of the research methodology and contributed to the data analysis and interpretation. All authors participated in the writing and revision of the article.

## Conflict of Interest

The authors declare that the research was conducted in the absence of any commercial or financial relationships that could be construed as a potential conflict of interest.
